# Associations between Dietary Nutrient Intakes and Hepatic Lipid Contents in NAFLD Patients Quantified by ^1^H-MRS and Dual-Echo MRI

**DOI:** 10.3390/nu8090527

**Published:** 2016-08-27

**Authors:** Yipeng Cheng, Kewei Zhang, Yang Chen, Yanchuan Li, Yuzheng Li, Kuang Fu, Rennan Feng

**Affiliations:** 1 Department of Magnetic Resonance Imaging, The Second Affiliated Hospital of Harbin Medical University, Harbin 150081, China; fanyun_strivecyp@sina.com; 2 Department of Mathematics, Heilongjiang Institute of Technology, Harbin 150027, China; zhangkewei2005@163.com; 3 Department of Nutrition and Food Hygiene, School of Public Health, Harbin Medical University, Harbin 150086, China; liushn0505@163.com (Y.C.); liyanchuan2013@foxmail.com (Y.L.); 15130143180@126.com (Y.L.)

**Keywords:** ^1^H-MRS, dual-echo MRI, dietary nutrient intakes, NAFLD

## Abstract

Dietary habits are crucial in the progression of hepatic lipid accumulation and nonalcoholic fatty liver disease (NAFLD). However, there are limited studies using ^1^H-magnetic resonance spectroscopy (^1^H-MRS) and dual-echo in-phase and out-phase magnetic resonance spectroscopy imaging (dual-echo MRI) to assess the effects of dietary nutrient intakes on hepatic lipid contents. In the present study, we recruited 36 female adults (NAFLD:control = 19:17) to receive questionnaires and medical examinations, including dietary intakes, anthropometric and biochemical measurements, and ^1^H-MRS and dual-echo MRI examinations. NAFLD patients were found to consume diets higher in energy, protein, fat, saturated fatty acid (SFA), and polyunsaturated fatty acid (PUFA). Total energy intake was positively associated with hepatic fat fraction (HFF) and intrahepatic lipid (IHL) after adjustment for age and body-mass index (BMI) (HFF: *β* = 0.24, *p* = 0.02; IHL: *β* = 0.38, *p* = 0.02). Total fat intake was positively associated with HFF and IHL after adjustment for age, BMI and total energy intake (HFF: *β* = 0.36, *p* = 0.03; IHL: *β* = 0.42, *p* = 0.01). SFA intake was positively associated with HFF and IHL after adjustments (HFF: *β* = 0.45, *p* = 0.003; IHL: *β* = 1.16, *p* = 0.03). In conclusion, hepatic fat content was associated with high energy, high fat and high SFA intakes, quantified by ^1^H-MRS and dual-echo MRI in our population. Our findings are useful to provide dietary targets to prevent the hepatic lipid accumulation and NAFLD.

## 1. Introduction

Nonalcoholic fatty liver disease (NAFLD) is characterized by the presence of hepatic lipid accumulation not due to secondary causes [1]. The prevalence of NAFLD ranges from 20% to 50% in most studies [1,2]. NAFLD is closely related with type-2 diabetes, cardiovascular disease, and hepatocellular carcinoma [3,4]. It has been established that lifestyle and dietary habits are all important contributors to NAFLD. High dietary fructose and trans-fatty acids are positively associated with NAFLD and NAFLD related diseases, including dyslipidemia, body fat deposition, and metabolic syndrome [5,6]. The increased intakes of monounsaturated fatty acids (MUFAs) and polyunsaturated fatty acids (PUFAs) exert beneficial effects on NAFLD patients [7,8]. MUFA and PUFA can lead to the decrease of plasma total cholesterol (TC) and low-density lipoprotein-cholesterol (LDL-C) [9]. PUFA can promote fatty acid oxidation by the up-regulation of genes related with fatty acid oxidation and prevent triglyceride storage by the down-regulation of genes related with lipid synthesis [7]. Thus, it is of great practical significance to study the relationship between dietary nutrient intakes and hepatic lipid accumulation to prevent NAFLD and related diseases.

Recently, ^1^H-magnetic resonance spectroscopy (^1^H-MRS) and dual-echo in-phase and out-phase magnetic resonance spectroscopy imaging (dual-echo MRI) have been widely reported in quantifying hepatic lipids accumulation in previous studies [10,11]. There are three ways to detect hepatic lipid accumulation, including ultrasonography, computed tomography and magnetic resonance imaging. A study found that hepatic ultrasonography had a low sensitivity to detect patients with <30% hepatic lipid accumulation [12]. Computer tomography requires that patients should be exposed to ionizing radiation. [13]. Nowadays, noninvasive measurements of ^1^H-MRS and dual-echo MRI are becoming important substitutes to liver biopsy in the diagnosis of NAFLD [14]. It has been illustrated that hepatic fat content measured by ^1^H-MRS and dual-echo MRI ranged from 0% to 50% and the hepatic fat content measured by MRI with fat suppression ranged from 0% to 100% [15]. The magnetic resonance imaging is regarded as the gold standard to detect the accumulation of liver fat because it can diagnose NAFLD patients with liver fat at levels below 7.5% [13,15]. It is the most accurate and effective technique in the evaluation of the hepatic lipids before liver biopsy. In addition, they have been illustrated to be correlated with biopsy results [13]. Owing to their sensitivity and specificity, we used ^1^H-MRS and dual-echo MRI to identify and quantify lipid contents in patients exposed to different dietary habits.

Considering all of the above, we used an internet-based diet and lifestyle questionnaire for Chinese (IDQC) to assess dietary nutrients intake and used ^1^H-MRS and dual-echo MRI to identify and quantify hepatic lipid contents. Finally, we analyzed the relationship between dietary nutrient intakes and hepatic lipid contents.

## 2. Methods

### 2.1. Participants and Demographic Characteristics

Female participants aged 30~65 year were enrolled at the Health Examination Center of the Second affiliated Hospital of Harbin Medical University from 1 April to 17 June 2015. Participants were instructed by staff to complete the online questionnaire and undertake medical examinations, including demographic data, dietary intakes, anthropometric and biochemical measurements, and ^1^H-MRS and dual-echo MRI examinations. Demographic data were collected using a standardized questionnaire online [16], including age, gender, physical activity at work (three groups: 1 = light, whose jobs had light physical activity, such as office staff, teachers, shop assistants, and so on; 2 = medium, whose jobs had regular physical activity, such as doctors, students, and so on; 3 = heavy, whose jobs had heavy physical activity, such as farmers, building workers, and so on), physical activity at leisure time (five groups: 0 = none, 1 = 1~30 min/week, 2 = 31~60 min/week, 3 = 61~90 min/week, 4 = 91~120 min/week and 5 ≥ 120 min/week), smoking (three groups: 1 = current smokers and 2 = non-smokers, 3 = quit smoking), and alcohol use (1 = current drinkers and 2 = non-drinkers, 3 = quit drinking).

The inclusion criteria were as follows: (1) female adults aged above 30 years; (2) stable body weight during the past four months (change in BMI < 0.5 kg/m^2^); (3) not taking any lipid, glucose, or blood pressure-lowering medications; and (4) not being pregnant. There were no significant differences in age, physical activity at work and leisure time, and menopause between the two groups. All participants gave written informed consents. Finally, a total of 36 individuals (NAFLD: control = 19:17) were randomly selected enroll the program. The study was approved by the Human Research Ethics Committee of the Harbin Medical University and complied with the Declaration of Helsinki (No. 2015001). At 90% statistical power and 5% significance level, a sample size of 13 in each group will be sufficient to detect a mean difference of 19.88% in IHL with a standard deviation of 10.71% between healthy adults and NAFLD patients.

### 2.2. Dietary Nutrients Intake Assessment

All participants were instructed to complete an IDQC, to record demographic data, lifestyles, and dietary habits for the most recent four months [17,18]. The absolute amounts of nutrient intakes were measured based on IDQC. The IDQC was a modern Food Frequency Questionnaire (FFQ) online, containing reference images of each food in different weights or volumes, which is beneficial to estimate the amount of food intake. Daily eaten foods contains 16 groups, including potatoes; grains; vegetables; fruits; beans and their products; fungus; seeds and nuts; livestock; poultry; dairy; eggs; fish; snacks; sweet foods; condiments; and beverages. The frequency of food intakes were divided into eight levels: never (<1 time/month), 1–3 times/month, 1 time/week, 2–3 times/week, 4–5 times/week, 1 time/day, 2 times/day, 3 or more than 3 times/day. The amount of food intakes were divided into 6 levels based on the daily proportion consumed: <1 liang (a Chinese traditional serving, 1 liang = 50 g), 2–3 liang, 4–5 liang, 6–7 liang, 8–9 liang, or more than 1 kg. For a small section of food, for instance, ginger, was divided into :<10 g, 20 to 30 g, 40 to 50 g, 60 to 70 g, 80 to 90 g, or >100 g. Beverages were counted as the volume per bottle (550 mL/bottle). The IDQC has been validated to be a reliable tool in dietary survey [16,19]. Nutrient contents of food were calculated according to China food composition tables, which were compiled and standardized by Peking University Medical Press, 2009 [20].

### 2.3. Anthropometric and Laboratory Measurements

Anthropometric parameters were measured twice by well-trained staff. If a second measurement was not close enough to the first measurement, a third measure was taken. Body weight, height, and waist circumference (WC) were measured to the nearest 0.1 kg and 0.1 cm wearing light clothes and no shoes. WC was measured mid-point between the lowest rib and the iliac crest using a flexible anthropometric tape in the horizontal plane. Body mass index (BMI) was calculated as weight in kilograms divided by height in meters squared. Systolic blood pressure (SBP) and diastolic blood pressure (DBP) were measured seated twice on the right arm using a standard mercury sphygmomanometer after a 5 min rest.

Peripheral venous blood samples were collected after fasting overnight. Antecubital venous blood was centrifuged to obtain serum and stored at −80 °C. The biochemical indicators were detected by a ROCHE Modular P800 Automatic Biochemical Analyzer (Roche Diagnostics, Mannheim, Germany), and included fasting blood glucose (FBG), total cholesterol (TC), triglycerides (TG), high-density lipoprotein cholesterol (HDL-C), low-density lipoprotein-cholesterol (LDL-C), aspartate aminotransferase (AST), alanine aminotransferase (ALT), alkaline phosphatase (ALP), serum creatinine (CRE), blood urea nitrogen (BUN) and serum uric acid (UA). Serum fasting insulin concentration was measured by a ROCHE Elecsys 2010 Chemiluminescence Immune Analyzer (Roche Diagnostics, Mannheim, Germany). The homeostasis model assessment of insulin resistance (HOMA-IR) index was calculated as previously described [21].

### 2.4. Definition of NAFLD

NAFLD was diagnosed based on the guidelines for NAFLD management formulated by the Chinese National Workshop on Fatty Liver Disease in 2010, as follows [22]: (1) alcohol consumption <140 g/week for male adults and <70 g/week for female adults; (2) absence of viral hepatitis [hepatitis B virus (HBV)/hepatitis C virus (HCV)], hepatolenticular degeneration, autoimmune diseases, a history of total parenteral nutrition, or intake of any hepatotoxic drugs (e.g., tamoxifen, amiodarone, sodium valproate, methotrexate, and glucocorticoid); and (3) ultrasonographic examination suggesting fatty infiltration in liver. The liver fatty infiltration was determined by the abdominal ultrasonographic examination using a 3.5-MHz probe (SSI-8000, Philips, The Netherlands). An experienced ultrasonographer, who was blind to the subjects’ disease history or blood laboratory analyses, conducted the examination. The liver condition was evaluated by size, contour, echogenicity, structure, and posterior beam attenuation.

### 2.5. Hepatic Lipid Contents Measurements

All participants underwent ^1^H-MRS and dual-echo MRI examinations. MR examination was performed on a 3.0-T scanner (Achieva TX, Philips Healthcare, Best, The Netherlands) with T2 correction. For in vivo ^1^H-MRS, a voxel was selected and shimmed after conventional imaging. Spectroscopy data were acquired with a point-resolved spectroscopy based on single-voxel (PRESS) technique; TR/TE, 2000/50; imaging time, 12 s; number of phase cycles, 4; spectral resolution, 1.95 Hz; without water suppression. Voxels (size 20 × 20 × 20 mm^3^) were placed in the right liver lobe trying to avoid bile ducts and larger vessels. Magnetic resonance spectra were analyzed with a PHILIPS Extended MR WorkSpace 2.6.3.2 that used an optimized set of basic functions to determine the relative concentrations of hepatic lipids. Intrahepatic lipid (IHL) was calculated as the formula: IHL = Slipid/(Slipid + Swater). Sfat was the area under the lipid peak (1.3 ppm) and Swater was the area under the water peak (4.7 ppm).

In this study, we also used the dual-echo MRI to assess the degree of lipid accumulation in liver. The transverse T1-weighted two-dimensional fast spoiled gradient-recalled (SPGR) dual-echo sequence was used for in phase/out of phase (IP/OP) imaging acquisition with the following parameters: TR, 180 ms; TE, 2.38 (opposed phase)/4.76 (in phase) ms; flip angle, 70; matrix size, 106 × 256; field of view, 37 cm. Briefly, three regions of interest (ROIs) were selected in the liver parenchyma areas for each image where there was no contamination from blood vessels and the sum of the numbers of pixels was at least 1000. The hepatic fat fraction (HFF; given in %) was calculated using the following formula: HFF = (Sin-Sout)/(2Sin) [23], where Sin and Sout were the signal intensity of IP and OP images, respectively.

### 2.6. Statistical Analysis

All continuous variables and categorical variables are separately presented as mean ± SD and the percentage. Data were tested for normality using Kolmogorov Smirnov test and abnormally-distributed variables were log transformed. Mean values for biochemical and dietary factors and categorical variables for demographics were compared separately by *t* test or chi-square test. Multivariate linear regression was used to assess the relationship between dietary variables and hepatic lipid contents. All statistical analyses were analyzed with SAS software (version 9.1; SAS Institute, Cary, NC, USA). *p* < 0.05 was considered statistically significant. The absolute amounts of nutrients were standardized to 100 kcal caloric intake to receive the relative consumption of nutrients.

## 3. Results

### 3.1. Characteristics and Anthropometric and Clinical Variables of Participants

The characteristics (age, physical activities at leisure time and work, menopause), and anthropometric and clinical variables are presented in Table 1. There were no significant differences in age, physical activities at leisure time and work, and menopause between NAFLD patients and healthy adults. Compared with healthy controls, NAFLD group showed significantly higher BMI, BFR, WC, and SBP. Additionally, serum insulin, TC, TG, LDL-C, ALT, and UA concentrations were significantly elevated in NAFLD patients. In addition, levels of HDL-C and HOMA-IR differed significantly.

**Figure 1:**
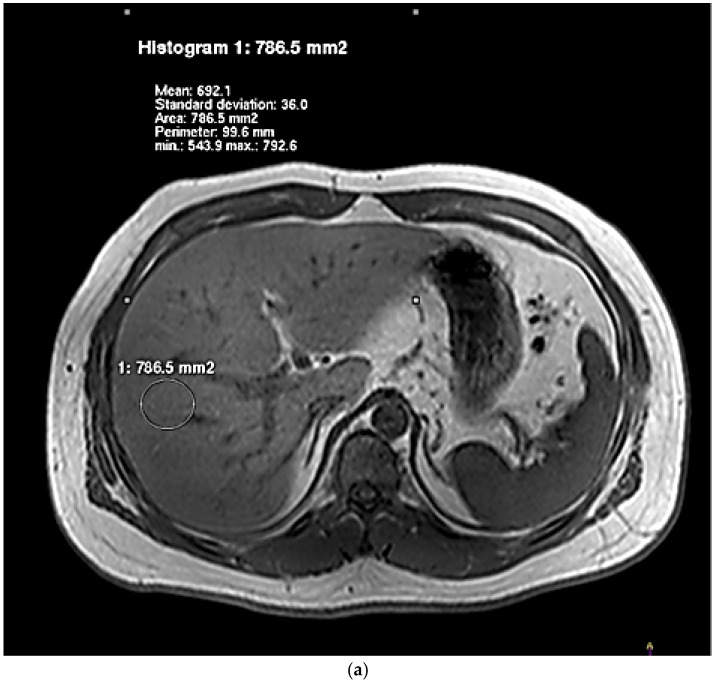

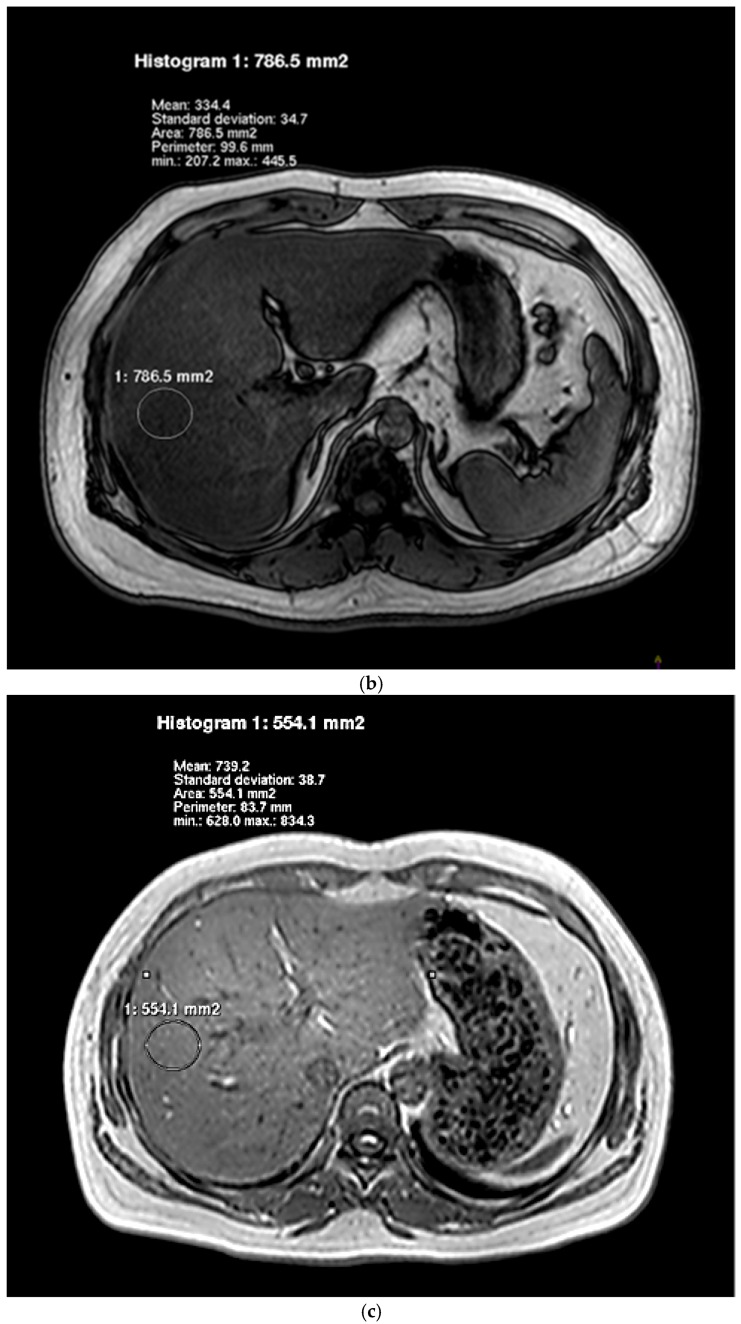

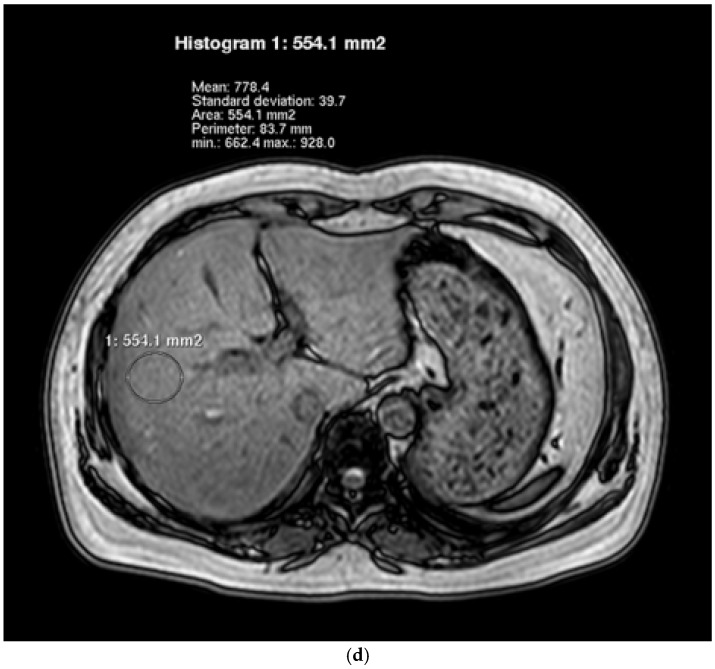
Representative IP (**a**,**c**) and OP (**b**,**d**) images of livers obtained by using dual-echo sequence. Images of a NAFLD patient and a healthy individual were, respectively, Figure 1a–d.

**Figure 2:**
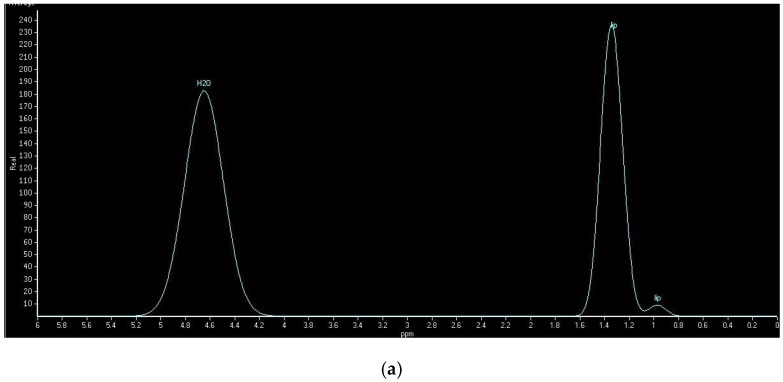

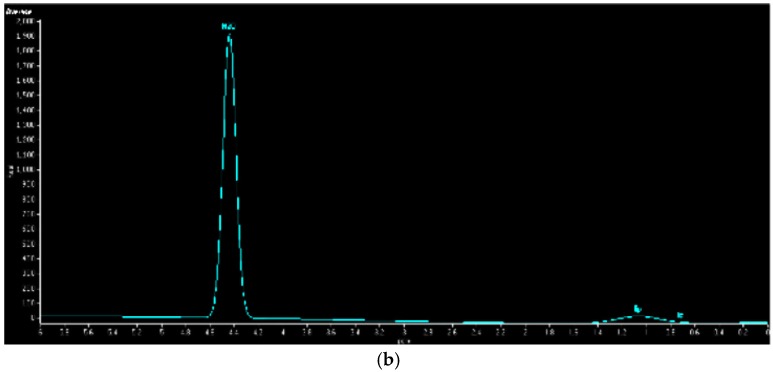
Typical spectra obtained from a NAFLD patient ((**a**), IHL = 36.1%) and a healthy individual ((**b**), IHL = 0.4%) showed the water signals and the lipid signals for calculating the IHL values.

The degree of hepatic lipid contents assessed by IHL and HFF is presented in Table 2. For the examination by dual-echo MRI, the fat and water signals within a voxel were, respectively, additive and subtractive in the in-phase image and the opposed-phase image (Figure 1). In the images of NAFLD patients, there was a decrease of the signal intensity from the in-phase to an opposed-phase image. NAFLD patients showed significantly higher HFF than controls. Figure 2 showed typical examples of ^1^H spectra for a NAFLD patient (A) and a control subject (B). NAFLD group had higher IHL, which contrasted sharply with controls.

### 3.2. Dietary Intakes of Participants

The dietary macronutrient intakes differed significantly between NAFLD patients and control group. Patients with NAFLD consumed more total energy, protein, and fat than controls (seen in Table 2). Then, we analyzed the differences of fat components intakes between NAFLD and controls. NAFLD patients ingested more saturated fatty acid (SFA) and PUFA than control group. The relative consumption of nutrients are shown in Table S1. NAFLD group showed significantly higher intake of fat per 100 kcal, low intake of carbohydrate per 100 kcal than the control group.

### 3.3. Associations of Dietary Nutrients and Hepatic Lipid Contents

Multivariate linear regression was used to analyze the associations between the absolute amounts of dietary nutrient intakes and hepatic lipid contents (Table 3). We firstly evaluated the impact of overall energy intake on hepatic lipid contents. We found that total energy intake was positively associated with both HFF and IHL with or without adjustments for age and BMI. For the dual-echo MRI examination, in the model 1 without adjustments, fiber and protein intakes were inversely associated with HFF, and total fat intake was positively associated with HFF (fiber: *β* = −0.29, *p* = 0.04; protein: *β* = −0.36, *p* = 0.01, total fat: *β* = 0.58, *p* = 0.005). After adjusting for age, total fat intake was positively associated with HFF (*β* = 0.31, *p* = 0.04). After adjusting for age and BMI, total fat intake was positively associated with HFF (*β* = 0.34, *p* = 0.03). Finally, we further adjusted for age, BMI, and total energy intake, total fat intake was positively associated with HFF (*β* = 0.36, *p* = 0.03). For the ^1^H-MRS examination, in the model 1 without adjustments, fiber and protein intakes were negatively associated with IHL, and carbohydrate and total fat intakes were positively associated with IHL (carbohydrate: *β* = 0.55, *p* = 0.01; total fat: *β* = 0.76, *p* = 0.003; fiber: *β* = −0.39, *p* = 0.04; protein: *β* = −0.65, *p* = 0.001). After adjusting for age, total fat intake was positively associated with IHL (*β* = 0.74, *p* < 0.001), while, after further adjusting for age and BMI, total fat intake was positively related with IHL (*β* = 0.49, *p* = 0.002). Finally, after adjusting for age, BMI, and total energy intake, total fat was positively related with IHL (*β* = 0.42, *p* = 0.01).

We also conducted the multivariate linear regression to analyze the relative consumption of nutrients and hepatic lipid contents (Table S2). For the dual-echo MRI examination, in the model 1 without adjustments, fiber per 100 kcal was inversely associated with HFF, and carbohydrate per 100 kcal and total fat per 100 kcal were positively associated with HFF (fiber per 100 kcal: *β* = −1.90, *p* = 0.02; carbohydrate per 100 kcal: *β* = 1.52, *p* = 0.03; total fat per 100 kcal: *β* = 2.22, *p* = 0.003). After adjusting for age, total fat per 100 kcal was positively associated with HFF (*β* = 2.23, *p* = 0.003). Finally, after adjusting for age and BMI, total fat per 100 kcal was positively associated with HFF (*β* = 1.47, *p* = 0.03). For the ^1^H-MRS examination, in the model 1 without adjustments, fiber per 100 kcal and protein per 100 kcal were inversely associated with IHL, and carbohydrate per 100 kcal and total fat per 100 kcal were positively associated with IHL (fiber per 100 kcal: *β* = −1.88, *p* = 0.03; carbohydrate per 100 kcal: *β* = 1.65, *p* = 0.02; total fat per 100 kcal: *β* = 2.75, *p* = 0.002). After adjusting for age, carbohydrate per 100 kcal, and total fat per 100 kcal were positively associated with IHL (carbohydrate per 100 kcal: *β* =1.68, *p* = 0.03; total fat per 100 kcal: *β* =2.77, *p* = 0.002). Finally, after adjusting for age and BMI, carbohydrate per 100 kcal and total fat per 100 kcal were both positively associated with IHL (carbohydrate per 100 kcal: *β* =0.98, *p* = 0.04; total fat per 100 kcal: *β* =1.24, *p* = 0.04).

Due to the strong correlation between the fat intake and hepatic lipid contents, we examined the relationship between SFA, PUFA, MUFA intakes and hepatic lipid contents (Table 4 and Table S3). We first conducted the multiple regression of the absolute consumption of fatty acids and hepatic lipid contents. For the dual-echo MRI examination, in model 1 without adjustments, SFA was positively correlated with HFF, and MUFA was negatively associated with HFF (SFA: *β* = 0.90, *p* = 0.008; MUFA: *β* = −0.96, *p* = 0.006). After adjustment for total energy intake, SFA was positively correlated with HFF, and MUFA was negatively associated with HFF (SFA: *β* = 0.76, *p* = 0.02; MUFA: *β* = −0.79, *p* = 0.03). While, after further adjustment for total energy intake and age, SFA was positively correlated with HFF (*β* = 0.72, *p* = 0.03). Finally, after adjustment for total energy intake, age and BMI, SFA was positively correlated with HFF (*β* = 0.45, *p* = 0.03). For the ^1^H-MRS examination, in the model 1 without adjustments, SFA was positively correlated with IHL, and MUFA and PUFA were negatively associated with IHL (SFA: *β* = 2.18, *p* = 0.007; MUFA: *β* = −0.74, *p* = 0.04; PUFA: *β* = −0.72, *p* = 0.02). After adjustment for total energy intake, SFA was positively correlated with IHL, and PUFA was negatively associated with IHL (SFA: *β* = 1.79, *p* = 0.02; PUFA: *β* = −0.72, *p* = 0.02), while, after further adjustment for total energy intake and age, SFA was positively correlated with IHL (*β* = 1.66, *p* = 0.03). Finally, after adjustment for total energy intake, age, and BMI, SFA was positively correlated with IHL (*β* = 1.16, *p* = 0.03).

We also analyzed the relative consumption of fatty acid intakes and hepatic lipid contents (Table S3). For the dual-echo MRI examination, in the model 1 without adjustments, SFA per 100 kcal was positively correlated with HFF, and MUFA and PUFA per 100 kcal were negatively associated with HFF (SFA per 100 kcal: *β* = 1.93, *p* = 0.02; MUFA per 100 kcal: *β* = −1.66, *p* = 0.01; PUFA per 100 kcal: *β* = −0.87, *p* = 0.001). After adjustment for total energy intake, SFA per 100 kcal was positively correlated with HFF, and PUFA was negatively associated with HFF (SFA per 100 kcal: *β* = 1.89, *p* = 0.03; PUFA per 100 kcal: *β* = −0.66, *p* = 0.01), while after further adjustment for total energy intake and age, SFA per 100 kcal was positively correlated with HFF (SFA per 100 kcal: *β* = 1.88, *p* = 0.03). Finally, after adjustment for total energy intake, age and BMI, SFA per 100 kcal was positively correlated with HFF (*β* = 1.55, *p* = 0.04). For the ^1^H-MRS examination, in model 1 without adjustments, SFA per 100 kcal was positively correlated with IHL, and MUFA and PUFA per 100 kcal were negatively associated with IHL (SFA per 100 kcal: *β* = 3.25, *p* = 0.004; MUFA per 100 kcal: *β* = −2.29, *p* = 0.002; PUFA per 100 kcal: *β* = −0.74, *p* = 0.01). After adjustment for total energy intake, SFA per 100 kcal was positively correlated with IHL, and MUFA per 100 kcal was negatively associated with IHL (SFA per 100 kcal: *β* = 3.21, *p* = 0.01; MUFA per 100 kcal: *β* = −1.77, *p* = 0.02), while after further adjustment for total energy intake and age, SFA per 100 kcal was positively correlated with IHL (SFA per 100 kcal: *β* = 2.51, *p* = 0.01). Finally, after adjustment for total energy intake, age and BMI, SFA per 100 kcal was positively correlated with IHL (SFA per 100 kcal: *β* = 1.93, *p* = 0.02).

## 4. Discussion

Our study is the first to use ^1^H-MRS and dual-echo MRI to assess the associations between dietary nutrient intakes and hepatic lipid contents in female NAFLD patients. We found that dietary fiber was negatively associated with both HFF and IHL without adjustments. MUFA and PUFA intakes were negatively associated with both HFF and IHL without adjustments. Total energy, total fat, and SFA intakes were positively associated with HFF and IHL after adjustments.

In our study, total energy intake was positively associated with both HFF and IHL after adjustments. Energy intake was positively associated with BMI z-score and WC, after controlled for confounders including physical activity and dietary bias [24]. A balanced diet with a weight reduction could improve hepatic inflammation and fat accumulation [25]. A study found that the most significant deviation between NAFLD and controls was the excessive energy intake [26]. However, the study did not evaluate the effect of nutrient intakes on hepatic lipid contents, which may weaken the association between dietary patterns and NAFLD. Furthermore, more and more NAFLD patients were found to have a normal BMI instead of obesity, which indicates that normal weight NAFLD patients may have unhealthy dietary patterns compared with controls [25]. All of the above results suggested that both energy and nutritional composition play vital roles in NAFLD.

In the current study, low fiber intake was positively associated with hepatic lipid contents. Fiber is beneficial for a series of health conditions including improving glucose, weight reduction and cardiovascular diseases [27] Previous studies showed that a diet high in fiber with a low-glycemic index was beneficial to decrease TC and improve insulin resistance, which may be helpful for NASH patients [28]. In addition, insulin resistance can result in the accumulation of lipids in liver [29]. Fiber is considered to increase gastric distension, increase satiety and, thus, decrease food intakes [30]. Soluble fiber can delay gastric emptying, thus decreasing the glucose absorption and serum insulin concentrations. This process also decreases the glucose available for hepatic lipogenesis, thus lowering serum lipids levels [31]. Insoluble fiber has bulking action and increases fecal mass [31,32]. Compared to soluble fiber, insoluble fiber is fermented to a lesser extent in the colon [32]. Overall, diets high in both soluble and insoluble fiber intakes may protect against insulin resistance and lipids accumulation in liver.

Previous studies showed that excessive intake of carbohydrates contributes to NAFLD [33]. Carbohydrates are the major substrate of de-novo lipogenesis in liver and, therefore, lead to hepatic lipid accumulation and the development of NAFLD [33,34]. However, the relationship between carbohydrate intake and hepatic lipid content in our study was not evident. The reason may be that the population ethnicity in our study is different (Caucasian vs. Chinese), the methods of quantifying hepatic lipids are different (ultrasonography vs. dual-echo MRI and 1H-MRS), and the carbohydrates we studied are all kinds, together. Further cohort studies are necessary to study the effect of carbohydrates in the pathogenesis of NAFLD.

We also found a positive correlation between total fat and hepatic lipid contents measured both by ^1^H-MRS and dual-echo MRI. Total fat intake was positively associated with both IHL and HFF with or without adjustments, which is consistent with previous studies [34]. High fat intake could contribute to the elevation of blood glucose, insulin, free fatty acids and TG concentrations [28,35]. The lipid accumulation makes liver exposed to high TG concentrations, which may disrupt the hepatic metabolic process [22]. Owing to the close relationship between total fat intake and hepatic lipid contents, we investigated the relationship of fatty acids and hepatic lipid contents in this study.

Our study showed that SFA intake was positively associated with both IHL and HFF in models with or without adjustments. A study comparing 25 NASH patients with age and BMI matched healthy individuals found that patients with NASH had higher intakes of SFA and lower PUFA than controls. They also found that SFA was positively related with insulin resistance and the postprandial rise of triglyceride [36]. SFA could promote endoplasmic reticulum (ER) stress and apoptosis in liver, whereas unsaturated fatty acids could reverse the damage of ER stress and apoptosis [37]. Data in our study found that MUFA and PUFA intakes were inversely associated with both IHL and HFF. Previous studies found the negative relationship of PUFA intake and liver fat measured by ultrasonography but not ^1^H-MRS and dual-echo MRI. Capanni et al. reported that liver echotexture measured by ultrasonography was improved after the increase of omega-3 PUFA intake [38]. Further, we also found that MUFA intake could be a protective factor for NAFLD. Ryan et al. instructed 12 individuals with NAFLD to undertake the Mediterranean diet high in MUFA, and found there was a significant reduction in patients with hepatic steatosis measured by ^1^H-MRS and an improvement of insulin sensitivity [8]. A diet high in MUFA and PUFA may improve insulin-resistant in NAFLD subjects by decreasing LDL cholesterol, and TG concentrations, and improving GLP-1 responses and peroxisome proliferator-activated receptor (PPARα) activity [8,38].

Our data also suggested that unhealthy dietary habits happened in the early stage of the development of NAFLD because serum liver aminotransferase concentration in our population was not beyond the normal range of clinical diagnosis (ALT concentration from 17 to 28 U/L; AST concentration from 14 to 42 U/L). It is implied that a healthy dietary pattern may prevent hepatic inflammation.

Our study gave insights into the associations of dietary intakes on hepatic lipid contents in female adults, NAFLD and other metabolic disorders. It emphasized that dietary factors played a vital role in the development of hepatic lipids accumulation and NAFLD. Limited sample size may influence the effect and further studies are needed to confirm this association. Due to the high prevalence of NAFLD in overweight/obese individuals [2], our study may provide dietary precautions against hepatic lipid accumulation and NAFLD in the general population.

## 5. Conclusions

We concluded that hepatic lipid content, quantified by ^1^H-MRS and dual-echo MRI, was associated with high intakes of energy, total fat, and SFA. Our findings might be useful to provide dietary targets to prevent the accumulation of hepatic lipid and NAFLD.

## Figures and Tables

**Table 1:** Characteristics and anthropometric and clinical variables of participants.

Characteristics	Control (*n* = 17)	NAFLD (*n* = 19)	*p* Value
Age (years)	48.76 ± 12.15	43.53 ± 10.94	0.18
Menopause (%)	35.29	26.32	0.82
Physical activity at leisure time (%)			0.24
0–30 min/week	11.76	15.79	
31–60 min/week	11.76	36.84	
61–120 min/week	52.94	26.32	
Above 120 min/week	23.53	21.05	
Physical activity at work (%)			0.55
Sedentary	76.47	89.47	
Moderate	23.53	10.53	
Heavy	0	0	
BMI (kg/m^2^)	26.84 ± 4.30	35.76 ± 4.53	<0.001
BFR (%)	35.63 ± 5.23	42.06 ± 3.55	<0.001
WC (cm)	95.90 ± 2.14	103.36 ± 7.86	0.001
SBP (mmHg)	102.50 ± 15.19	120.63 ± 16.73	<0.001
DBP (mmHg)	84.07 ± 15.78	92.89 ± 8.21	0.06
FBG (mmol/L)	5.17 ± 0.81	5.58 ± 1.13	0.22
Insulin (mU/L)	6.48 ± 0.69	18.23 ± 7.15	<0.001
HOMA-IR	1.49 ± 0.30	4.65 ± 2.38	<0.001
TC (mmol/L)	4.20 ± 0.39	5.15 ± 0.61	<0.001
TG (mmol/L)	0.67 ± 0.20	1.69 ± 1.14	<0.001
HDL-C (mmol/L)	1.64 ± 0.32	1.36 ± 0.16	0.002
LDL-C (mmol/L)	1.98 ± 0.34	3.03 ± 0.68	<0.001
ALT (U/L)	15.71 ± 2.42	23.35 ± 11.94	0.02
AST (U/L)	18.06 ± 1.98	21.33 ± 6.95	0.07
ALP (U/L)	43.00 ± 13.22	46.05 ± 9.99	0.44
BUN (mmol/L)	4.79 ± 1.28	5.22 ± 1.56	0.39
CRE (µmol/L)	49.88 ± 8.13	55.89 ± 11.78	0.09
UA (µmol/L)	239.24 ± 20.37	339.91 ± 75.13	<0.001
HFF (%)	5.22 ± 1.27	20.13 ± 1.12	<0.001
IHL (%)	3.54 ± 1.81	23.42 ± 4.78	<0.001

All values are shown as means ± SD for continuous variable and categorical variables are the percentage of participants. Independent *t* tests and chi-square tests were separately used to compare differences in continuous variables and categorical variables. If not normally distributed after transformation, a Mann-Whitney U test was conducted. BMI, body mass index; BFR, body fat ratio; WC, waist circumference; SBP, systolic blood pressure; DBP, diastolic blood pressure; FBG, fasting blood glucose; HOMA-IR, homeostasis model assessment of insulin resistance; TC, total cholesterol; TG, triglycerides; HDL-C, high-density lipoprotein cholesterol; LDL-C, low-density lipoprotein-cholesterol; ALT, alanine aminotransferase; AST, aspartate aminotransferase; ALP, alkaline phosphatase; BUN, blood urea nitrogen; CRE, creatinine; UA, uric acid; HFF, hepatic fat fraction; IHL, intrahepatic lipid.

**Table 2:** Dietary intakes of participants.

Variables	Controls (*n* = 17)	NAFLD (*n* = 19)	*p* Value
Energy (Kcal)	2423.74 ± 431.08	2901.00 ± 682.74	0.02
Protein (g)	79.17 ± 17.72	96.77 ± 22.59	0.01
Fat (g)	60.35 ± 16.33	86.56 ± 18.26	<0.001
Carbohydrate (g) ^§^	390.98 ± 83.33	433.73 ± 117.72	0.22
Fiber (g)	19.67 ± 5.47	21.45 ± 6.21	0.37
SFA (g)	6.13 ± 1.42	8.13 ± 2.31	<0.001
MUFA (g) ^§^	8.80 ± 3.84	10.78 ± 3.04	0.09
PUFA (g) ^§^	11.2 ± 4.78	15.95 ± 5.24	0.01

All continuous variables are presented as means ± SD. ^§^ Abnormally distributed variables were log transformed. Independent *t* tests were used to compare differences in continuous variables. SFA, saturated fatty acid, PUFA, polyunsaturated fatty acid; MUFA, monounsaturated fatty acid.

**Table 3:** Associations between dietary nutrient intakes and HFF and IHL.

Variables in Model	HFF (%, *n* = 36)		IHL (%, *n* = 36)	
	*β*	*p*	*β*	*p*
Carbohydrate				
Model 1	0.11	0.25	0.55	0.01
Model 2	0.05	0.52	0.04	0.38
Model 3	0.02	0.37	0.31	0.07
Model 4	0.02	0.41	0.17	0.15
Fiber				
Model 1	−0.29	0.04	−0.39	0.04
Model 2	−0.24	0.07	−0.09	0.23
Model 3	−0.16	0.08	−0.08	0.35
Model 4	−0.13	0.10	−0.05	0.40
Protein				
Model 1	−0.36	0.01	−0.65	0.001
Model 2	−0.03	0.56	−0.38	0.06
Model 3	−0.01	0.39	−0.13	0.11
Model 4	−0.01	0.41	−0.11	0.14
Total fat				
Model 1	0.58	0.005	0.76	0.003
Model 2	0.31	0.04	0.74	<0.001
Model 3	0.34	0.03	0.49	0.002
Model 4	0.36	0.03	0.42	0.01
Total energy				
Model 5	0.35	0.01	0.47	0.004
Model 6	0.34	0.02	0.49	0.01
Model 7	0.24	0.02	0.38	0.02

Model 1 contained carbohydrate, fiber, protein, and total fat with no adjustments; Model 2 contained the same macronutrients with adjustment for age; Model 3 contained the same macronutrients with adjustment for age and BMI; Model 4 contained the same macronutrients with adjustment for age, BMI, and total energy; Model 5 contained total energy without adjustments; Model 6 contained total energy with adjustment for age; Model 7 contained total energy with adjustment for age and BMI. HFF, hepatic fat fraction; IHL, intrahepatic lipid.

**Table 4:** Associations between fatty acids intake and HFF and IHL.

Variables in Model	HFF (%, *n* = 36)		IHL (%, *n* = 36)	
	*β*	*p*	*β*	*p*
SFA				
Model 1	0.90	0.008	2.18	0.007
Model 2	0.76	0.02	1.79	0.02
Model 3	0.72	0.03	1.66	0.03
Model 4	0.45	0.03	1.16	0.03
MUFA				
Model 1	−0.96	0.006	−0.74	0.04
Model 2	−0.79	0.03	−0.59	0.05
Model 3	−0.41	0.16	−0.54	0.06
Model 4	−0.39	0.19	−0.51	0.07
PUFA				
Model 1	−2.38	0.05	−0.72	0.02
Model 2	−2.32	0.05	−0.72	0.02
Model 3	−1.39	0.06	−0.06	0.08
Model 4	−1.19	0.10	−0.10	0.07

Model 1 was unadjusted. Model 2 was adjusted for total energy intake; Model 3 was adjusted for total energy intake and age; Model 4 was adjusted for total energy intake, age, and BMI. SFA, saturated fatty acid, PUFA, polyunsaturated fatty acid; MUFA, monounsaturated fatty acid; HFF, hepatic fat fraction; IHL, intrahepatic lipid.

## Supplementary Materials

The following are available online at http://www.mdpi.com/2072-6643/8/9/527/s1, Table S1: Dietary standardized values of the nutrients, Table S2: Associations between dietary standardized values of nutrients and HFF and IHL, Table S3: Associations between fatty acids standardized values and HFF and IHL.

## Abbreviations

H-MRS	^1^H-magnetic resonance spectroscopy
Dual-echo MRI	Dual-echo in-phase and out-phase magnetic resonance spectroscopy imaging
NAFLD	nonalcoholic fatty liver disease
IDQC	internet-based diet and lifestyle questionnaire for Chinese
SFA	saturated fatty acid
PUFA	polyunsaturated fatty acid
HFF	hepatic fat fraction
IHL	intrahepatic lipid
MUFA	monounsaturated fatty acid
