# Supplementary material for: Associations between Dietary Nutrient Intakes and Hepatic Lipid Contents in NAFLD Patients Quantified by 1H-MRS and Dual-Echo MRI
Source: Nutrients. 2016 Aug 27;8(9):527. doi: 10.3390/nu8090527 (PMC5037514; doi:10.3390/nu8090527)
Supplement: Supplementary file 1 [file nutrients-08-00527-s001.docx]

Supplementary Materials: Associations between Dietary Nutrient Intakes and Hepatic Lipid Contents in NAFLD Patients Quantified by ^1^H-MRS and
Dual-Echo MRI

Yipeng Cheng, Kewei Zhang, Yang Chen, Yanchuan Li, Yuzheng Li, Kuang Fu and Rennan Feng

**Table S1.** Dietary standardized values of the nutrients.

| **Variables** | **Controls (*n* = 17)** | **NAFLD (*n* = 19)** | ***p* Value** |
| --- | --- | --- | --- |
| Protein (g)/100 kcal | 3.26 ± 0.43 | 3.34 ± 0.24 | 0.49 |
| Fat (g)/100 kcal | 2.50 ± 0.59 | 3.02 ± 0.47 | 0.01 |
| Carbohydrate (g)/100 kcal | 16.12 ± 1.68 | 14.85 ± 1.24 | 0.02 |
| Fiber (g)/100 kcal | 0.81 ± 0.19 | 0.75 ± 0.21 | 0.40 |
| SFA (g)/100 kcal | 0.26 ± 0.07 | 0.30 ± 0.12 | 0.17 |
| MUFA (g)/100 kcal | 0.37 ± 0.17 | 0.39 ± 0.15 | 0.70 |
| PUFA (g)/100 kcal | 0.47 ± 0.20 | 0.59 ± 0.27 | 0.12 |

All continuous variables are presented as means ± SD. Independent t-tests were used to compare differences in continuous variables. SFA, saturated fatty acid, PUFA, polyunsaturated fatty acid; MUFA, monounsaturated fatty acid.

**Table S2.** Associations between dietary standardized values of nutrients and HFF and IHL.

| **Variables in Model** | **HFF (%, *n* = 36)** | | **IHL (%, *n* = 36)** | |
| --- | --- | --- | --- | --- |
|  | ***β*** | ***p*** | ***β*** | ***p*** |
| Carbohydrate (g)/100 kcal | |  |  |  |
| Model 1 | 1.52 | 0.03 | 1.65 | 0.02 |
| Model 2 | 1.63 | 0.06 | 1.68 | 0.03 |
| Model 3 | 0.97 | 0.09 | 0.98 | 0.04 |
| Fiber (g)/100 kcal |  |  |  |  |
| Model 1 | −1.90 | 0.02 | −1.88 | 0.03 |
| Model 2 | −0.26 | 0.39 | −0.23 | 0.40 |
| Model 3 | −0.11 | 0.44 | −0.06 | 0.47 |
| Protein (g)/100 kcal |  |  |  |  |
| Model 1 | −1.13 | 0.07 | −1.21 | 0.05 |
| Model 2 | −1.01 | 0.09 | −1.13 | 0.06 |
| Model 3 | −0.58 | 0.21 | −0.02 | 0.98 |
| Total fat (g)/100 kcal |  |  |  |  |
| Model 1 | 2.22 | 0.003 | 2.75 | 0.002 |
| Model 2 | 2.23 | 0.003 | 2.77 | 0.002 |
| Model 3 | 1.47 | 0.03 | 1.24 | 0.04 |

Model 1 contained carbohydrate (g)/100 kcal, fiber (g)/100 kcal, protein (g)/100 kcal, total fat (g)/100 kcal with no adjustments; Model 2 contained the same variables with adjustment for age; Model 3 contained the same variables with adjustment for age and BMI. HFF, hepatic fat fraction; IHL, intrahepatic lipid.

**Table S3.** Associations between fatty acids standardized values and HFF and IHL.

| **Variables in Model** | **HFF (%, *n* = 36)** | | **IHL (%, *n* = 36)** | |
| --- | --- | --- | --- | --- |
|  | ***β*** | ***p*** | ***β*** | ***p*** |
| SFA (g)/100 kcal | |  |  |  |
| Model 1 | 1.93 | 0.02 | 3.25 | 0.004 |
| Model 2 | 1.89 | 0.03 | 3.21 | 0.01 |
| Model 3 | 1.88 | 0.03 | 2.51 | 0.01 |
| Model 4 | 1.55 | 0.04 | 1.93 | 0.02 |
| MUFA (g)/100 kcal | |  |  |  |
| Model 1 | −1.66 | 0.01 | −2.29 | 0.002 |
| Model 2 | −1.22 | 0.05 | −1.77 | 0.02 |
| Model 3 | −0.41 | 0.43 | −0.92 | 0.15 |
| Model 4 | −0.95 | 0.20 | −1.41 | 0.12 |
| PUFA (g)/100 kcal | |  |  |  |
| Model 1 | −0.87 | 0.001 | −0.74 | 0.01 |
| Model 2 | −0.66 | 0.01 | −0.49 | 0.09 |
| Model 3 | −0.55 | 0.07 | −0.35 | 0.17 |
| Model 4 | −0.08 | 0.42 | −0.15 | 0.31 |

Model 1 was unadjusted. Model 2 was adjusted for total energy intake; Model 3 was adjusted for total energy intake and age; Model 4 was adjusted for total energy intake, age and BMI. HFF, hepatic fat fraction; IHL, intrahepatic lipid.
